# The PD-Ballet study: study protocol for a randomised controlled single-blind hybrid type 2 clinical trial evaluating the effects of ballet dancing on motor and non-motor symptoms in Parkinson’s disease

**DOI:** 10.1186/s12906-023-04296-y

**Published:** 2024-01-17

**Authors:** Aleksandra M. Podlewska, Lucia Batzu, Tayana Soukup, Nick Sevdalis, Ioannis Bakolis, Fleur Derbyshire-Fox, Alison Hartley, Andy Healey, Anthony Woods, Nikki Crane, Carmine Pariante, K Ray Chaudhuri

**Affiliations:** 1 King’s College London, Institute of Psychiatry, Psychology and Neuroscience, London, UK; 2 Parkinson’s Foundation Centre of Excellence at King’s College Hospital, London, UK; 3 Imperial College London, Faculty of Medicine, London, UK; 4 National University of Singapore, Yong Loo Lin School of Medicine, Singapore, Singapore; 5 English National Ballet, London, UK

**Keywords:** Parkinson’s disease, Non-motor symptoms, Dance, Exercise, Intervention, RCT, Programmed exercise

## Abstract

**Background:**

To date, beneficial effects of multimodal exercise programmes on Parkinson’s disease (PD) have focused on motor symptoms and little attention has been paid to the potential effects of such programmes on the non-motor symptoms of PD, which are now universally known as one of the key drivers of quality of life and a key unmet need. We aim to explore clinical effectiveness of a ballet-based dance programme in addressing non-motor and motor symptoms of Parkinson’s disease across all stages of progression.

**Methods:**

A randomised, single-blind, controlled trial of 160 people with Parkinson’s across all motor stages (Participants will be stratified into three groups of motor advancement: Hoehn and Yahr (HY) stages I and II being Mild Group, HY Stage III being Moderate Group and HY Stages IV and V being Severe Group) will be randomly allocated to either an intervention or a control group using an independent randomisation body. The primary outcome is an improvement in non-motor symptoms as measured by the Movement Disorders Society Non-Motor Scale (MDS-NMS). The intervention protocol consists of 12 one-weekly dance sessions led by English National Ballet. Each session is followed by a ‘tea and biscuit’ social time. Control group follows standard clinical pathway and joins the ‘tea and biscuit’ to control for any positive effects of social interactions. All participants are assessed at baseline, immediately after completion of the intervention and 3–6 months later to explore any potential longitudinal effects.

**Discussion:**

To our knowledge, no adequately powered study has explored the effects of a dance-based intervention on non-motor symptoms of Parkinson’s disease, assessing these on both holistic and granular levels. We also aim to stratify participants in accordance with their motor state as assessed by.

HY staging to explore specific effects on the symptoms at the initial, moderate and complex stages of the disease. If successful, this trial provides first evidence on clinical effectiveness of a ballet-based dance intervention for symptoms of Parkinson’s disease, assessed in a robust, rigorous manner.

**Trial registration:**

NCT04719468.

## Administrative information

**Table:** 

Title {1}	The PD-Ballet study: a randomised controlled single-blind hybrid type 2 clinical trial evaluating the effects of ballet dancing on motor and non-motor symptoms in Parkinson’s disease
Trial registration {2a and 2b}	NCT04719468 IRAS: 275,588
Protocol version {3}	V4 dated 01/03/2022
Funding {4}	Wellcome Trust (grant code: RE16059)
Author details {5a}	Aleksandra M Podlewska: Department of Basic and Clinical Neuroscience, Institute of Psychiatry, Psychology & Neuroscience, King's College London, London SE5 9RT, UK; Parkinson's Foundation Centre of Excellence, King's College Hospital, London SE5 9RS, UK; aleksandra.podlewska@kcl.ac.uk Lucia Batzu: Department of Basic and Clinical Neuroscience, Institute of Psychiatry, Psychology & Neuroscience, King's College London, London SE5 9RT, UK; Parkinson's Foundation Centre of Excellence, King's College Hospital, London SE5 9RS, UK; lucia.batzu@kcl.ac.uk Tayana Soukup: Faculty of Medicine, Department of Surgery and Cancer, Hammersmith Hospital, London W12 0HS, UK; t.soukup@imperial.ac.uk Nick Sevdalis: Centre for Behavioural & Implementation Science Interventions, National University of Singapore, Yong Loo Lin School of Medicine, Clinical Research Centre, 117, 597 Singapore, Singapore; nick.sevdalis04@gmail.com Ioannis Bakolis: Department of Biostatistics and Health Informatics, Institute of Psychiatry, Psychology and Neuroscience, King’s College London, David Goldberg Centre, 18 De Crespigny Park, London, SE5 8AF, UK; ioannis.bakolis@kcl.ac.uk Fleur Derbyshire-Fox: Participant Engagement, English National Ballet, 41 Hopewell Square, London, E14 0SY, UK; Fleur.Derbyshire-Fox@ballet.org.uk Alison Hartley: Participant Engagement, English National Ballet, 41 Hopewell Square, London, E14 0SY, UK; alison.hartley@ballet.org.uk Andy Healey: Department of Biostatistics and Health Informatics, Institute of Psychiatry, Psychology and Neuroscience, King’s College London, David Goldberg Centre, 18 De Crespigny Park, London, SE5 8AF, UK; andy.healey@kcl.ac.uk Anthony Woods: Department of Basic and Clinical Neuroscience, Institute of Psychiatry, Psychology & Neuroscience, King's College London, London SE5 9RT, UK; tony.woods@kcl.ac.uk Nikki Crane: Department of Basic and Clinical Neuroscience, Institute of Psychiatry, Psychology & Neuroscience, King's College London, London SE5 9RT, UK; nikki.crane@kcl.ac.uk Carmine Pariante: Department of Basic and Clinical Neuroscience, Institute of Psychiatry, Psychology & Neuroscience, King's College London, London SE5 9RT, UK; carmine.pariante@kcl.ac.uk K Ray Chaudhuri: Department of Basic and Clinical Neuroscience, Institute of Psychiatry, Psychology & Neuroscience, King's College London, London SE5 9RT, UK; Parkinson's Foundation Centre of Excellence, King's College Hospital, London SE5 9RS, UK; ray.chaudhuri@kcl.ac.uk
Name and contact information for the trial sponsor {5b}	Professor Raza Razavi Director of Administration (Health Schools). Room 5.31, James Clerk Maxwell Building, 57 Waterloo Road, London, SE1 8WA
Role of sponsor {5c}	King’s College London in co-sponsorship with King’s College Hospital NHS Foundation Trust. Responsible for the initiation, management and arranging of the financing of the trial. The institutions carry medico-legal responsibility associated with its conduct

## Introduction

### Background and rationale

Parkinson’s Disease (PD) is the second most common neurodegenerative condition, with over 127,000 people having this diagnosis only in the United Kingdom and a rising prevalence, likely to double by 2050 [1]. PD is a multisystem progressive disorder characterised by a range of motor symptoms including, but not limited to, rigidity, bradykinesia, rest tremor and gait dysfunction. Non-motor symptoms (NMS) such as sleep disturbance, drooling and cognitive decline have been characterised by James Parkinson himself [2] with more recent literature outlining that the majority of the most bothersome symptoms reported by people with Parkinson’s (PwP) are, in fact, non-motor [3]. Yet, current treatment of the disease, which is mostly pharmacological and heavily reliant on dopaminergic therapies, is still largely focused on the motor symptoms, with most areas in NMS being largely unaddressed and remaining an unmet need [4].

A range of non-pharmacological management strategies have been proposed to be of benefit in PD and exercise has been demonstrated as beneficial in various chronic diseases including stroke, diabetes, dementia and osteoporosis [5] and is increasingly advocated as an adjunct intervention for individuals with PD [6]. Recent literature shows that exercise-based interventions have a positive impact on physical and functional capacities as well as quality of life, with some evidence pointing towards neuroprotective effects [7–9].

NMS such as mood dysfunctions, pain, sleep problems, fatigue, apathy and gastrointestinal issues are all reported as symptoms imposing major burden with deteriorating quality of life on PwP [10, 11]. Given the previous evidence for exercise being beneficial in addressing these symptoms, the current study will aim to explore the possible benefits of a dance-based exercise therapy for NMS.

In 2009, English National Ballet (ENB) developed a community model of dance sessions for PwP drawing its strengths as a world-class ballet company, which has an established circuit touring nationally. The model, initially tested in London, has proven to be replicable and has been trialled in four other locations throughout the UK—Oxford, Cardiff, Ipswich and Liverpool. Previous research on this programme examined the effects on the body, on activities of daily living and made links to social participation as well as the artistic engagement by participants. The research demonstrated that the ENB’s Dance for Parkinson’s is of value to participants, particularly emotionally, socially, and artistically and therefore, touching on several aspects of the spectrum of nonmotor symptoms of PD [12].

Nevertheless, the number of randomised clinical trials of dance investigating gains in both motor and non-motor symptoms remains low. To date, little focus was placed on ballet-based dance as an intervention for PD. Equally, there is no research using wearable sensors for the assessment of motor gains following a dance-based intervention. Our research aims to address this important gap in the evidence base. As part of a wider project exploring the effects of art interventions in health, SHAPER [13], we propose a randomised controlled clinical trial investigating the effectiveness of ballet dancing as an adjuvant therapy in PD, as assessed by a range of validated clinical outcome measures and objective wearable sensors. The PD-Ballet study is a hybrid trial (type II, [14]) investigating both clinical effectiveness and implementation, and the current protocol manuscript will outline the clinical effectiveness evaluation; the implementation effectiveness evaluation is discussed in separate protocol [15].

## Objectives

The main trial objectives of the study are two-fold and given its hybrid type II model, the study has clinical effectiveness as well as implementation science as primary objectives which are outlined separately [15]. The primary objective for clinical effectiveness is to evaluate the effects of the interventions on the non-motor symptoms of PD, as defined by the change to the total score of the Movement Disorders Society-sponsored Non-Motor Scale (MDS-NMS) [16].

## Trial design

PD-Ballet is a randomised, controlled, single blind hybrid type II clinical trial performed at a single site (King’s College Hospital NHS Foundation Trust).

This is a hybrid type II effectiveness-implementation trial, where we place equal focus on the effectiveness of the intervention and the effectiveness of its implementation. Hybrid effectiveness-implementation evaluations are considered the gold standard of modern implementation science and blend design components of clinical effectiveness and implementation research [17]. Such blending provides more rapid translational gains, better guidance for intervention adaptation and effective implementation, and more useful information for decision makers, thereby reducing wastage in research [17].

The current manuscript will outline the study design focusing on the clinical effectiveness.

Study participants will be stratified into one of the three motor groups (mild, moderate and severe based on accepted Hoehn and Yahr (HY) stage-based scores [18], see Table 3) and then randomly assigned to either the intervention or the control group in a 2:1 ratio. The main outcome measure is MDS-NMS. Study raters will be blinded to the participant condition allocation. Study assessments will be carried out at baseline, end of the intervention and 3–6 months later, in accordance with participant availability. A separate Implementation science protocol has been published [15].

## Methods: participants, interventions and outcomes

### Study setting

Study assessments will be carried out at the Clinical Research Facility within King’s College Hospital, London, UK. The intervention will be carried out at the ENB School of Dance in London.

### Eligibility criteria

Participants must be willing and able to attend the weekly dance sessions led by English National Ballet dance artists and the weekly “tea and biscuit” social sessions taking place after the dance sessions. Participants allocated to the control group will be provided with a virtual platform link to join the ‘tea and biscuit’ sessions remotely, to minimise the travel burden and the unnecessary risk of infection with SARS-CoV-2, though they are also invited to attend the ‘tea and biscuit’ sessions in person, personal circumstances permitting. Furthermore, eligible participants will be over 18 years old and have a confirmed diagnosis of idiopathic PD with a HY stage I to V (in ‘ON’ state) [18]. Both drug-naïve and medicated patients will be eligible for participation. Patients who are under consideration for advanced therapies will not be eligible for participation. All medicated participants must be on a stable medication regimen for at least one month prior to baseline assessments. For all study participants, optimal treatment will be kept stable, though on demand changes will be allowed based on clinician discretion; this will be noted in the case report forms.

In summary:

- Inclusion criteria:
  age of 18 and upwards;
  diagnosis of idiopathic PD according to the MDS Clinical Diagnostic Criteria for Parkinson’s disease 2015 [19];
  HY stages I-V.
- Exclusion criteria:
  diagnosis or suspicion of other causes for parkinsonism;
  advanced-stage therapy consideration (deep brain stimulation, continuous levodopa duodenal infusion, and continuous subcutaneous apomorphine infusion);
  any condition interfering with the ability to give the informed consent;
  indication of dementia through a score of ≤ 21 on the Montreal Cognitive Assessment (MoCA) [20];
  enrolment in a simultaneous investigational trial;
  inability to attend to the weekly sessions.

### Who will take informed consent?

Informed consent will be sought prior to any study activities and will be gained in line with Good Clinical Practice (GCP) and to the ethical principles that have their origin in the Declaration of Helsinki. All researchers delegated to obtain informed consent will have an up to date GCP training.

### Additional consent provisions for collection and use of participant data and biological specimens

Separate consent will be sought for the implementation science data collection, as outlined in the implementation science study protocol [15].

## Interventions

### Explanation for the choice of comparators

The intervention will be delivered by the ENB dancers. Participants in the control group will follow the standard treatment per the local pathway and at the discretion of the lead physician and will attend the social ‘tea and biscuit’ sessions to control for any effects of the social interactions.

### Intervention description

The intervention programme will be delivered within a dance studio and led by four specialist dance artists and musicians trained by ENB, with support of assistants. Participants will engage in weekly classes, incorporating live music, dance, rhythm and voice. Artistic content is inspired by ENB’s classical and contemporary works and will provide a framework for participants to explore narrative, characters, themes, concepts and music. A seated or standing warm-up will involve stretches of the torso in all directions, a focus on breathing and a balanced posture, followed by emphasised movement of the lower body and rhythmic exercises to challenge coordination. Musicians and dance artists will work together to introduce various vocal warm-ups inspired by the artistic theme to introduce another layer of expression and intention. The class will develop from the barre to the centre with travelling dance phrases either with a partner or in formation, followed by structured improvisation tasks and the learning and development of repertoire dance phrases. All dance material is adapted as necessary for each individual to participate fully. Each dance session will comprise one hour and fifteen minutes of the activity, followed by “tea and biscuits” social time and refreshments for up to one hour. The total duration of the intervention will be 12 weeks for both the active and the control groups. The intervention will be delivered by the same dance artists and supporting staff across the three groups.

### Criteria for discontinuing or modifying allocated interventions

Participants will discontinue the active intervention should they experience adverse events (AEs) or serious adverse events (SAEs) preventing them from participation in the dance sessions. No other factors are anticipated to prevent completion of the allocated intervention.

### Strategies to improve adherence to interventions

Adherence will be measured based on attrition rate and number of missed sessions for each participant. Adherence to the intervention will be encouraged by the research team: each week, participants will be contacted in order to ensure availability of transport to arrive to the intervention venue, as well as by the ENB team.

### Relevant concomitant care permitted or prohibited during the trial

Participants’ general practitioners will be notified of study involvement and participants will be encouraged to keep their antiparkinsonian medication regimen stable for one month prior to, and during the intervention. Any changes will be noted in the case report forms and taken into account during statistical analysis. Participants who will commence on an advanced therapy during the course of the trial will be excluded.

### Provisions for post-trial care

Participants allocated to the control group will be offered the intervention on a compassionate basis once all study activities are complete. All participants will have a follow-up visit with the study team three to six months after completion of the intervention to monitor for any AEs or other events as well as any changes to the condition.

### Outcomes

Assessments will be performed at baseline, post-intervention and at three to six months follow-up, depending on the participant availability. All assessments will be performed in the ON state to minimise any possible motor-related limitations as well as the risk of off-related non-motor fluctuations [18, 19]. For each participant, assessments at all timepoints will be performed at the same time of day to ensure consistency in their ON state performance. Clinical outcome measures are summarised in Table 1. Objective motor measure using the Parkinson’s KinetiGraph (PKG™) will be performed continuously for six days prior to the clinical assessments.

**Table 1:** Outline of clinical outcome measures

Investigator-reported outcomes (patient-focused)	Patient reported outcomes (PROs)	Caregiver reported outcomes
1. MDS-Unified Parkinson’s Disease Rating Scale (MDS-UPDRS) [21] parts I, II, III and IV 2. MDS-Non-motor symptoms scale (MDS-NMS) [16] 3. 10-m walk test [22] 4. King’s Parkinson’s Pain Scale (KPPS) [23] 5. Timed Up and Go test 6. Montreal Cognitive Assessment (MoCA) [24] 7. Clinical Impression of Severity Index for Parkinson’s disease (CISI-PD) [25]	1. Parkinson’s Disease Sleep Scale 2 (PDSS-2) [26] 2. Parkinson’s Disease Questionnaire-8 (PDQ-8) [27] 3. Hospital Anxiety and Depression Scale (HADS) [28] 4. Schwab and England Scale [29] 5. EQ-5D-5L questionnaire [30] 6. Parkinson’s Fatigue Scale-16 (PFS-16) [31] 7. Physical Activity Scale for the Elderly (PASE) [32, 33] 8. Starkstein Apathy Scale [34] 9. Wearing Off Questionnaire-9 (107) 10. Gastrointestinal Dysfunction Scale for Parkinson’s Disease (GIDS-PD) [35]	1. Zarit Burden Interview [36]

### Primary clinical effectiveness outcome measure

The primary outcome measure will be the difference in the total score on the Movement Disorders Society Non-Motor Scale (MDS-NMS) at baseline and the end-of-intervention assessments. MDS-NMS is a 52-item Movement Disorders Society-sponsored revision of the original Non-Motor Symptom Scale (NMSS) [10], validated for clinical and research use and to measure the burden of non-motor symptoms and non-motor fluctuations in PD patients. The score for a minimal clinically important change of this new instrument is based on the widely used NMSS [37, 38] which has been extensively used in clinical practice and trials. For MDS-NMS, both inter- and intra-rater validity is high, though to minimise any possible rater bias, the same, blinded rater will perform assessments at all three time points for each participant.

### Secondary clinical effectiveness outcome measures

#### Motor symptoms

To explore the effects of the intervention on the daily motor function of the participants, the Movement-Disorders Society sponsored revision of the Unified Parkinson’s Disease Rating Scale Part III (MDS-UPDRS-III) [21, 39] will be performed at all three time points. MDS-UPDRS is the most widely used tool to assess PD, consisting of four parts, with part III focusing on the assessment of the motor function. For an objective measure of motor symptoms including bradykinesia, dyskinesia, tremor and time spent immobile, a Parkinson KinetiGraph (PKG) wearable sensor [40] will be worn by the participants continuously for six days at baseline and end-of-intervention assessments. PKG is a clinically validated objective tool providing scores for bradykinesia and dyskinesia and a percentage of time spent with tremor and immobile. Other motor assessments performed in clinic will include 10-Meter Walk test (10MWT) [41] and Timed up and Go test (TUG) [24]. 10MWT is a validated tool measuring the speed of gait in PD. The difference in meters covered per second both at leisurely and fast paces will be measured at all three time points. The TUG will be used to measure patients’ ability to perform sequential motor tasks. The difference in the total time it takes to perform the task at all time points (in seconds) will be measured. Motor fluctuations will be measured using the MDS-UPDRS part IV and falls including near falls will be noted retrospectively at each study assessment during the clinical interview.

#### Non-motor symptoms

We will use a mixture of clinician-based and patient-completed outcome measures to assess these changes. Given that exercise has been reported to have beneficial effects on cognitive functioning in PD [42], cognition will be assessed using the MoCA. The tool has been validated for use in Parkinson’s disease and is demonstrated to be more sensitive to temporal changes than the Mini Mental State Examination [43].

Changes to mood, including symptoms of depression and anxiety will be assessed using a 14-item patient-completed Hospital Anxiety and Depression Scale (HADS), validated and sensitive in PD [28]. Fatigue, increasingly more reported as a problem in PD, will be assessed using the Parkinson’s Fatigue Scale (PFS-16) [31], a 16-item self-completed scale recommended by the MDS Task Force [44].

To ensure that changes to fatigue and sleep-related symptoms are differentiated, we will also employ Parkinson’s Disease Sleep Scale 2 (PDSS-2) [45], a 15-item self-reported tool, widely used both in clinical practice and research in PD. Apathy, another important NMS in PD, will be assessed using the well-validated and widely used, self-rated 14-item Apathy Scale (AS) [34, 46].

Wearing off, a well-reported phenomenon in PD, will be assessed using the motor fluctuations questions in Part IV of the MDS-UPDRS, and using the patient-completed, 9-item Wearing-off Questionnaire [47]. The non-motor wearing off phenomenon will be assessed using the Non-Motor Fluctuations (NMF) subscale of the MDS-NMS.

We will also evaluate the possible beneficial effect of the intervention on constipation, given that gastrointestinal dysfunction is a common problem in PD, especially at the later stages of the disease. We will utilise the Gastrointestinal Dysfunction Scale for Parkinson’s Disease (GIDS-PD) [35], a validated patient-completed questionnaire specifically assessing gastrointestinal dysfunction, including constipation in PD.

Finally, to assess the effects of the intervention on pain, one of the most underappreciated symptoms in PD, we will employ the King’s Parkinson’s Pain Scale (KPPS); a 14-item clinician-completed comprehensive tool assessing 7 different domains of pain in PD [23].

#### Quality of life

Quality of life and the ability to perform activities of daily living, as well as the clinical impression of change following the intervention will also be explored. A shortened, patient-rated 8-item version of the Parkinson’s Disease Questionnaire (PDQ-8) will be employed alongside the EuroQol (EQ-5D-5L) questionnaire [27, 30, 48], both validated for use in PD to assess health-related quality of life. Clinicians will also complete the Clinical Impression of Severity Index (CISI-PD) [25] and patients will be asked to rate their independence using the Schwab and England Scale [49], rated from 0 (completely dependent) to 100% (completely independent). In addition, reduced independence in activities of daily living requires assistance from carers, and as such, we will also measure the level of burden experienced by the carers using the Zarit Burden Interview, a 22-item questionnaire validated for carers for PwP [36, 50, 51].

#### Physical activity

All study participants will also complete the Physical Activity Scale for the Elderly (PASE) [32, 52] in order to control for pre-existing levels of physical activity. This is a validated, patient-completed scale assessing the intensity, frequency and duration of activity over the past week.

A parallel Implementation measures will be employed, as outlined in a separate study protocol [15].

### Participant timeline

**Fig. 1:**
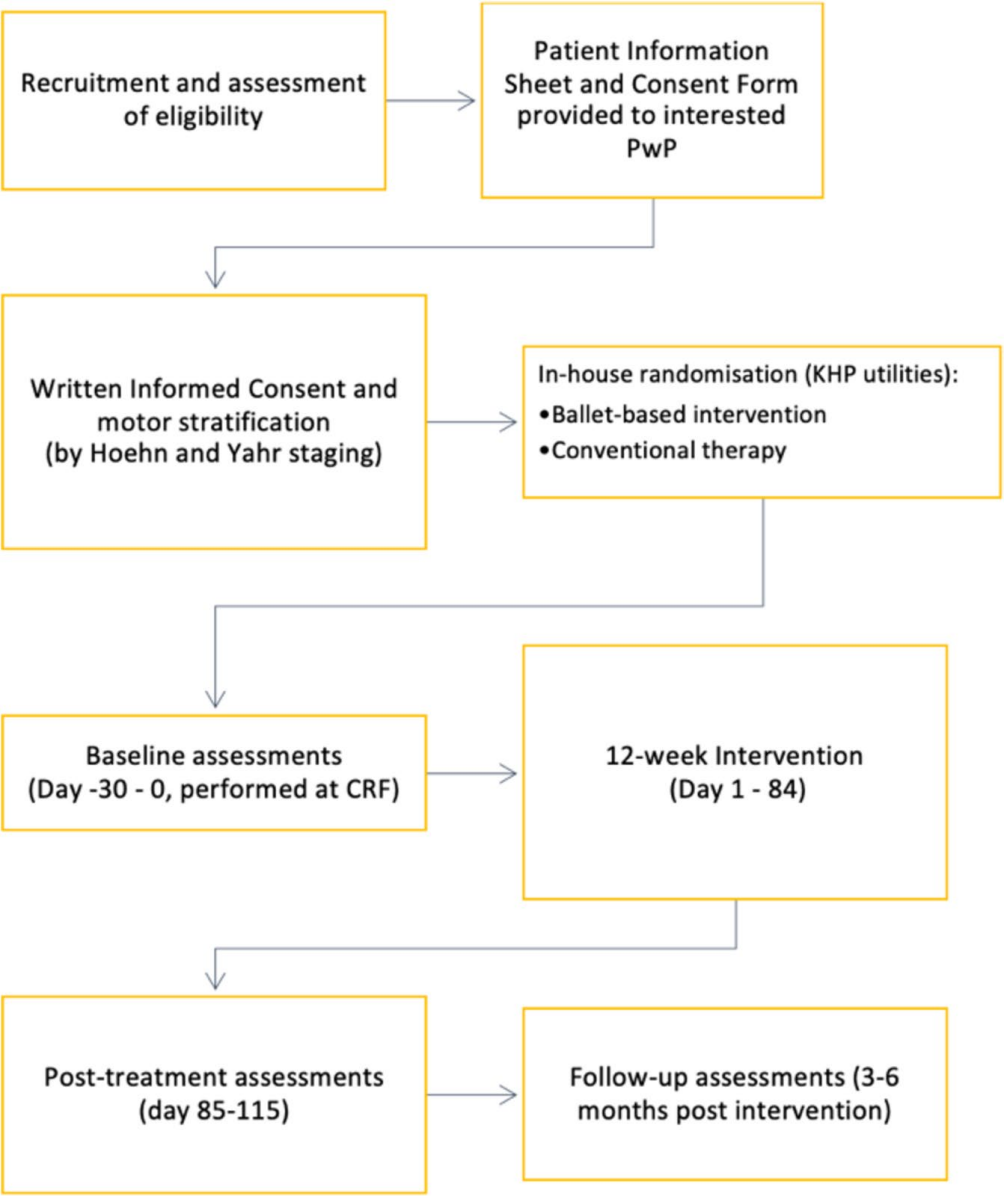
Participant timeline

Figure 1 outlines participant timeline and Table 2 illustrates the study assessment schedule.

**Table 2:** Study assessment schedule

Study assessment schedule	Baseline	Immediately post intervention	3–6 months post intervention
Demographic and clinical characteristics	✓	✓	✓
Falls questionnaire	✓	✓	✓
Parkinson’s KinetiGraph recording^a^	✓	✓	✓
Validated questionnaires^b^	✓	✓	✓
Health Care Resource Utilisation Form	✓	✓	✓
Compliance		✓	

^**a**^Performed within ≤ 30 days prior to baseline and post intervention. ^b^The following scales will be completed at week 6: MDS-UPDRS Part II and PDQ-8 (participants will be sent these by post/electronically to complete at home and return to the study team)

### Sample size

Given that no previous studies explored the effect of ballet dancing on the symptoms of PD, sample size calculation is based on detecting a significant difference between intervention and the control group in the mean changes from baseline to follow-up in the primary outcome (MDS-NMS). To date, no clinical trials have yet utilised MDS-NMS as an outcome measure due to its novelty. As such, our estimation of the sample size is based on detecting a medium effect size (Cohen’s d 0.5) with 80% power and 5% significance, resulting in the total of 144 participants. Given that the randomisation ratio is 2:1, the intervention group is expected to include 96 participants and the control group 48 participants. Allowing a 10% attrition rate for the final analysis, the total number of participants in the study will be 160. The study is powered as a superiority study at immediate follow-up.

### Recruitment

**Fig. 2:**
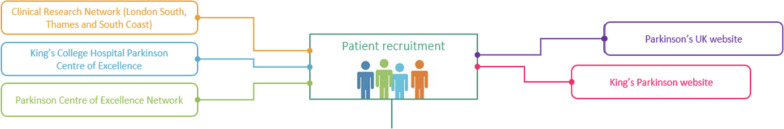
Recruitment strategies

Participants will be recruited mainly through the regional, national, and tertiary Movement Disorders outpatient clinics at the Parkinson’s Foundation Centre of Excellence, King’s College Hospital NHS Foundation Trust, London, United Kingdom. The study will be advertised via the South London Clinical Research Network, Parkinson’s UK Centre of Excellence Network, King’s Parkinson’s website (www.parkinsons-london.co.uk) and the corresponding social media, as well as the English National Ballet network. Recruitment strategies are outlined in Fig. 2. Recruitment period was originally planned to last for 12 months, but due to Covid-19 restrictions, this has been extended to 18 months. Participation eligibility will be determined at screening, with a subsequent assessment of motor advancement to ensure appropriate group allocation (mild, moderate or severe motor PD) (Table 3).

**Table 3:** Stratification of study participants

Group 1: mild	Hoehn and Yahr stages I and II
Group 2: moderate	Hoehn and Yahr stage III
Group 3: severe	Hoehn and Yahr stages IV and V

## Assignment of interventions: allocation

### Sequence generation

Following motor advancement stratification, participants will be randomly assigned to one of the two arms (intervention or control group) using an in-house, electronic randomisation system provided by King’s Health Partners, an independent body providing support for studies conducted at King’s College Hospital.

### Concealment mechanism

After informed consent and baseline data collection, participants will be randomly divided into one of the two arms at a ratio of 2:1, intervention and control group specifically. Participant allocations will be kept concealed with an unblinded study coordinator, who will contact all participants will allocations prior to commencement of the intervention. Study raters will remain blinded.

### Implementation

All procedures will be implemented in accordance with the protocol approved by the NIHR Research Ethics Committee. A local Research and Development department will ensure appropriate protocol implementation and monitoring. Additionally, the Research Ethics Committee requires an annual progress report to ensure appropriate implementation of the study protocol and safety oversight.

## Assignment of interventions: blinding

### Who will be blinded

Study personnel will consist of blinded raters and unblinded coordinators who will be in communication with the ENB coordinators to ensure that the right participants are enrolled onto the appropriate sessions (intervention and social session as opposed to social session only). Medical oversight will be provided by unblinded neurologist. Data analyst will remain blinded.

### Procedure for unblinding if needed

N/A due to the fact that there is an unblinded medical oversight.

## Data collection and management

### Plans for assessment and collection of outcomes

Prior to any data collection, all study personnel will be trained on the relevant standard operating procedures implemented locally at study sites. All personnel will also complete GCP training and, where applicable, blinded raters will be trained on the use of study assessments, including the MDS-UPDRS and MDS-NMS certifications. Blinded raters will be kept consistent across study visits to minimise inter-rater variability. Where possible, electronic data collection will be enabled to minimise participant burden during the study visits on site. For patient-completed outcomes, all participants will be sent a participant-reported case report forms to complete at home on the day of the in-clinic study visits. An independent data monitoring will be utilised to ensure transparency and traceability. All data will be entered onto an electronic data capture designed and maintained by the Clinical Trials Unit at King’s Health Partners. On study completion and upon query resolution, the study database will be locked and validated for analysis.

### Plans to promote participant retention and complete follow-up

There are no specific plans to promote participant retention.

### Data management

All data will be managed by the research team and monitored by an independent coordinator. Study data will be entered onto electronic database and checked for validity and accuracy. Upon all query resolution, the database will be locked for analysis. Any changes to the locked database will be agreed upon by the trial statistician, data manager and the chief investigator.

### Confidentiality

Study team will have access to confidential information including medical notes for study participants. Once enrolled, all participants will be assigned a study identifier (ID) and all case report forms will only bear participant study ID. No personal data will be shared with individuals outside of the study team. Upon trial completion, all participant data will be fully anonymised before statistical analyses. All procedures pertaining to anonymisation have been approved by the Research Ethics Committee.

### Plans for collection, laboratory evaluation and storage of biological specimens for genetic or molecular analysis in this trial/future use

N/A. No biological samples will be collected.

## Statistical methods

### Statistical methods for primary and secondary outcomes

The first stage of analyses will be a descriptive model of the data to assess completeness of data and the integrity of the data collection system. Participant and centre characteristics and demographics will be summarised at baseline. Clinical characteristics that have been measured repeatedly will be summarised at baseline and at the post intervention follow-up assessments. In addition, patterns of missing data will be described.

The primary clinical outcome will be analysed using linear mixed effects models, to model the difference in means in total score of MDS-NMS between the two arms (Ballet vs Standard therapy) at immediate post intervention follow up. Linear mixed effects models will be adjusted for baseline total score of MDS-NMS and the severity of PD (HY staging). A two-level hierarchical model will be employed when all time points will be included as repeated measures in the model (post intervention and 6 months) to improve power and take into account clustering of the observation at patient level. These models utilise maximum likelihood estimation and thus allow for missing outcome data under the missing at random (MAR) assumption. Associations between post-randomisation variables and missingness will be dealt with by multiple imputation (MI), again under the MAR assumption. Departures from this assumption will be assessed with a sensitivity analysis. Secondary outcomes will be assessed with a similar methodology for the primary outcomes, using generalized linear mixed models depending on the type of outcome (normal, binary, count). All the questionnaires to be used would have validated methods of scoring, and the scores will be analysed as described.

### Interim analyses

N/A. No interim analyses is planned.

### Methods for additional analyses (e.g. subgroup analyses)

N/A.

#### Methods in analysis to handle protocol non-adherence and any statistical methods to handle missing data

The analyses will be primarily based on intention to treat (ITT); a per protocol (PP) analysis will be the secondary analysis to be carried out. The PP analysis includes participants who undertake the stipulated interventions (adherence = 75%), comparing the intervention effectiveness and will exclude any participant who was non-adherent based on non-attendance of more than 3 sessions (75%).

Statistical methods to handle missing data have been outlined in the section Statistical methods for primary and secondary outcomes {20a}.

### Plans to give access to the full protocol, participant level-data and statistical code

Full protocol, participant-level data and statistical code will be made available to researchers upon request deemed reasonable.

## Oversight and monitoring

### Composition of the coordinating centre and trial steering committee

The Trial Steering Committee (TCS) will include the Chief Investigator, study investigators, data manager, a statistician and trial manager. TSC will meet annually prior to the submission of the annual progress report to the Research Ethics Committee.

### Composition of the data monitoring committee, its role and reporting structure

The TSC will be responsible for data monitoring, along with the quality assurance officer working within the Research and Development department at King’s College Hospital NHS Foundation Trust.

### Adverse event reporting and harms

Adverse events (AE) and serious adverse events (SAE) will be collected for all study participants. Causality will be assessed by the unblinded neurologist. All AEs and SAEs will be reported in accordance to the local standard operating procedures. AEs will be reportable within 28 calendar days and SAEs will be reportable within 24 h. All reports will be made to the study sponsor and where required, to the Research Ethics Committee.

### Frequency and plans for auditing trial conduct

Trial conduct will be audited by the quality assurance officer working within the Research and Development department at King’s College Hospital NHS Foundation Trust.

### Plans for communicating important protocol amendments to relevant parties (e.g. trial participants, ethical committees)

All amendments to the protocol or patient-facing material will be reviewed by the NHIR Research Ethics Committee. Where required, study participants will be made aware of the amendments. All relevant study personnel will be informed of the changes. Where applicable, protocol version control and review of amendments will be documented for each study team member and signed off by the Chief Investigator.

### Dissemination plans

Study results will be submitted for publication within 12 months of the publishing of the final trial report. Data will be presented at conferences and meetings and published in peer-reviewed journals. All findings will be disseminated to the study participants in the form of a follow-up newsletter.

## Discussion

We hereby present the rationale and the design of the PD-Ballet study, a hybrid type II effectiveness-implementation trial. Several studies have now focused on the effects of exercise on the symptoms of PD, though to date and to our knowledge, no adequately powered study has explored the effects of a dance-based intervention on NMS, assessing these on both holistic and granular levels. We have deliberately chosen to utilise the novel adaptation of the original NMSS, the MDS-NMS, to match the current standards for assessment in clinical trials for PD.

The SARS-CoV-2 pandemic has had a major impact on deliverability of the intervention and, while a remote delivery had been considered, the national lockdowns are no longer in place and we aim to test the effects of the intervention in its original form, which has been previously demonstrated as feasible and safe to deliver.

We aim to stratify participants in accordance with their motor state as assessed by the HY staging in order to explore specific effects on the NMS at the initial, moderate and complex stages of the disease.

We accept that there might be cultural bias pertaining to ballet being largely considered as destined for Caucasian, middle to upper class individuals, and we intend to pay specific attention to recruitment of diverse populations, given the recent evidence demonstrating diversity shortcomings in clinical trials within PD populations [53]. To address this problem, we collaborate with a Patient and Public Involvement-led Focus Group [54] to improve our understanding of the possible barriers to recruitment and develop culturally bespoke pathways to increase awareness and understanding of ballet-based dancing interventions across culturally different PwP. Additionally, together with ENB, we developed the roles of PwP Champions, who help promote the study across their social and cultural circles to aid awareness and increase interest in learning more about the aims of the intervention.

### Trial status

The total trial period has been extended to 31 March 2024 and is expected to be extended thereafter to ensure all data collection is completed. Patient recruitment began on 01 March 2022 and is currently underway. Protocol version number and date: V4.0, dated 1st March 2022. All recruitment is expected to be completed by 1st December 2023.

## Data Availability

Final datasets will be available to the study team and researchers contributing to the analyses, under strict data sharing agreements in place, which are governed by the study sponsor.

## Abbreviations

10MWT: 10-Meter walk test
AE: Adverse event
CISI-PD: Clinical impression of severity index for parkinson’s disease
CRISP: Community for research involvement and support by PwPs
ENB: English National Ballet
GCP: Good clinical practice
GIDS-PD: Gastrointestinal dysfunction scale for Parkinson’s disease
HADS: Hospital anxiety and depression scale
HY: Hoehn and Yahr stage
KPPS: King’s Parkinson’s disease pain scale
MDS: Movement Disorders Society
MDS-NMS: Movement Disorders Society Non-motor Symptom Scale
MDS-UPDRS: Movement disorders society sponsored unified PD rating scale
MoCA: Montreal cognitive assessment
NMF: Non-motor fluctuations
NMS: Non-motor symptoms
NMSS: Non-motor symptoms scale
PASE: Physical activity scale for the elderly
PD: Parkinson’s disease
PDQ-8: Parkinson’s disease questionnaire-8
PDSS-2: Parkinson’s disease sleep scale 2
PFS-16: Parkinson’s fatigue scale-16
PKG: Parkinson’s Kinetigraph
PwP: People with Parkinson’s
SAE: Serious adverse event
TUG: Timed up and go
